# A general model of hippocampal and dorsal striatal learning and decision making

**DOI:** 10.1073/pnas.2007981117

**Published:** 2020-11-23

**Authors:** Jesse P. Geerts, Fabian Chersi, Kimberly L. Stachenfeld, Neil Burgess

**Affiliations:** ^a^Sainsbury Wellcome Centre for Neural Circuits and Behaviour, University College London, London W1T 4JG, United Kingdom;; ^b^Institute of Cognitive Neuroscience, University College London, London WC1N 3AZ, United Kingdom;; ^c^GrAI Matter Labs, 75012 Paris, France;; ^d^DeepMind, London N1C 4AG, United Kingdom

**Keywords:** reinforcement learning, spatial navigation, hippocampus, striatum

## Abstract

Humans and other animals use multiple strategies for making decisions. Reinforcement-learning theory distinguishes between stimulus–response (model-free; MF) learning and deliberative (model-based; MB) planning. The spatial-navigation literature presents a parallel dichotomy between navigation strategies. In “response learning,” associated with the dorsolateral striatum (DLS), decisions are anchored to an egocentric reference frame. In “place learning,” associated with the hippocampus, decisions are anchored to an allocentric reference frame. Emerging evidence suggests that the contribution of hippocampus to place learning may also underlie its contribution to MB learning by representing relational structure in a cognitive map. Here, we introduce a computational model in which hippocampus subserves place and MB learning by learning a “successor representation” of relational structure between states; DLS implements model-free response learning by learning associations between actions and egocentric representations of landmarks; and action values from either system are weighted by the reliability of its predictions. We show that this model reproduces a range of seemingly disparate behavioral findings in spatial and nonspatial decision tasks and explains the effects of lesions to DLS and hippocampus on these tasks. Furthermore, modeling place cells as driven by boundaries explains the observation that, unlike navigation guided by landmarks, navigation guided by boundaries is robust to “blocking” by prior state–reward associations due to learned associations between place cells. Our model, originally shaped by detailed constraints in the spatial literature, successfully characterizes the hippocampal–striatal system as a general system for decision making via adaptive combination of stimulus–response learning and the use of a cognitive map.

## Significance

A central question in neuroscience concerns how humans and animals trade off multiple decision-making strategies. Another question pertains to the use of egocentric and allocentric strategies during navigation. We introduce reinforcement-learning models based on learning to predict future reward directly from states and actions or via learning to predict future “successor” states, choosing actions from either system based on the reliability of its predictions. We show that this model explains behavior on both spatial and nonspatial decision tasks, and we map the two model components onto the function of the dorsal hippocampus and the dorsolateral striatum, thereby unifying findings from the spatial-navigation and decision-making fields.

Behavioral and neuroscientific studies suggest that animals can apply multiple strategies to the problem of maximizing future reward, referred to as the reinforcement-learning (RL) problem (1, 2). One strategy is to build a model of the environment that can be used to simulate the future to plan optimal actions (3) and the past for episodic memory (4–6). An alternative, model-free (MF) approach uses trial and error to estimate a direct mapping from the animal’s state to its expected future reward, which the agent caches and looks up at decision time (7, 8), potentially supporting procedural memory (9). This computation is thought to be carried out in the brain through prediction errors signaled by phasic dopamine responses (10). These strategies are associated with different tradeoffs (2). The model-based (MB) approach is powerful and flexible, but computationally expensive and, therefore, slow at decision time. MF methods, in contrast, enable rapid action selection, but these methods learn slowly and adapt poorly to changing environments. In addition to MF and MB methods, there are intermediate solutions that rely on learning useful representations that reduce burdens on the downstream RL process (11–13).

In the spatial-memory literature, a distinction has been observed between “response learning” and “place learning” (14–16). When navigating to a previously visited location, response learning involves learning a sequence of actions, each of which depends on the preceding action or sensory cue (expressed in egocentric terms). For example, one might remember a sequence of left and right turns starting from a specific landmark. An alternative place-learning strategy involves learning a flexible internal representation of the spatial layout of the environment (expressed in allocentric terms). This “cognitive map” is thought to be supported by the hippocampal formation, where there are neurons tuned to place and heading direction (17–19). Spatial navigation using this map is flexible because it can be used with arbitrary starting locations and destinations, which need not be marked by immediate sensory cues.

We posit that the distinction between place and response learning is analogous to that between MB and MF RL (20). Under this view, associative reinforcement is supported by the DLS (21, 22). Indeed, there is evidence from both rodents (23–25) and humans (26, 27) that spatial-response learning relies on the same basal ganglia structures that support MF RL. Evidence also suggests an analogy between MB reasoning and hippocampus (HPC)-based place learning (28, 29). However, this equivalence is not completely straightforward. For example, in rodents, multiple hippocampal lesion and inactivation studies failed to elicit an effect on action-outcome learning, a hallmark of MB planning (30–35). Nevertheless, there are indications that HPC might contribute to a different aspect of MB RL: namely, the representation of relational structure. Tasks that require memory of the relationships between stimuli do show dependence on HPC (36–42).

Here, we formalize the perspective that hippocampal contributions to MB learning and place learning are the same, as are the dorsolateral striatal contributions to MF and response learning. In our model, HPC supports flexible behavior by representing the relational structure among different allocentric states, while dorsolateral striatum (DLS) supports associative reinforcement over egocentric sensory features. The model arbitrates between the use of these systems by weighting each system’s action values by the reliability of the system, as measured by a recent average of prediction errors, following Wan Lee et al. (43). We show that HPC and DLS maintain these roles across multiple task domains, including a range of spatial and nonspatial tasks. Our model can quantitatively explain a range of seemingly disparate findings, including the choice between place and response strategies in spatial navigation (23, 44) and choices on nonspatial multistep decision tasks (45, 46). Furthermore, it explains the puzzling finding that landmark-guided navigation is sensitive to the blocking effect, whereas boundary-guided navigation is not (27), and that these are supported by the DLS and HPC, respectively (26). Thus, different RL strategies that manage competing tradeoffs can explain a longstanding body of spatial navigation and decision-making literature under a unified model.

## Results

**Fig. 1.**
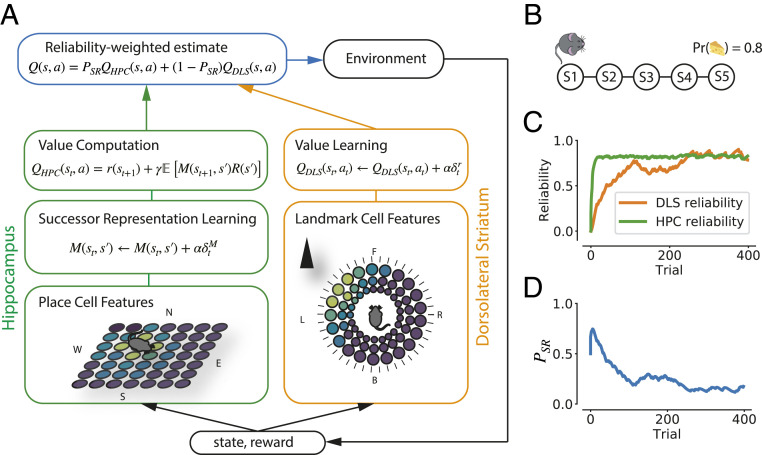
(*A*) Model architecture. DLS (orange) learns value directly from landmark features in egocentric directions with respect to the agent: L (left), R (right), F (front), or B (back). HPC (green) learns an SR M over allocentric input features (north, N; east, E; south, S; or west, W), which is subsequently used for value computation. An arbitrator (blue) computes an average of these values, weighted by each system’s reliability (*Materials and Methods*). Lighter colors mean higher firing rates. α, learning rate; $\delta^{M}$, SPE; $\delta^{r}$, reward-prediction error; $P_{HPC}$, proportion of influence of HPC component. (*B*) A linear track environment with five states. Terminal state S5 gives a reward with probability 0.8. (*C*) Reliability of the hippocampal SR system and the striatal MF system over time as the agent navigates the linear track. Reliability is computed based on the recent average of SPEs $\delta^{M}$ for the hippocampal system- and reward-prediction errors $\delta^{R}$ for the striatal system. (*D*) The proportion of influence of the SR system on the value function, $P_{SR}$, in the linear track environment across trials.

We implemented a model of hippocampal and dorsolateral striatal contributions to learning, shown in Fig. 1. Each system independently proposes an action and estimates its value. The value Q(s,a) of taking action a while being in state s is the expected discounted cumulative return:

$$Q\left(s,a\right)=E_{\pi }\left[\overset{\infty }{\underset{t=0}{\sum }}\gamma^{t}r\left(s_{t}\right)|s_{0}=s,a_{0}=a\right],$$ [1]

where $s_{0}$ and $a_{0}$ are the starting state and action at time t=0, r is a reward function specifying the instantaneous reward found in each state, γ∈[0,1) is a discount factor that gives smaller weight to distal rewards, and π(a|s) is the policy specifying a distribution over available actions given the current state. The objective of the RL agent is to discover an optimal policy $\pi^{*}$ that will maximize value over all states.

Similarly to earlier work in spatial RL (15, 47–49), the two systems in our model estimate value using qualitatively different strategies, which can cause them to generate divergent predictions for the optimal policy. The dorsal striatal component uses an MF temporal difference (TD) method (50) to learn stimulus–response associations directly from egocentric sensory inputs given by landmark cells (LCs) tuned to landmarks at given distances and egocentric directions from the agent (Fig. 1*A* and *Materials and Methods*).

The hippocampal component, in contrast, has access to state information provided by place cells that, in spatial tasks, fire when the agent occupies specific locations. We draw on previous work by Stachenfeld et al. (51) and model hippocampal place cells as encoding the successor representation (SR; ref. 11). The SR is a predictive representation, containing the discounted future occupancy of each state $s^{\prime }$ from current state s:

$$M^{\pi }\left(s,s^{\prime }\right)=E_{\pi }\left[\overset{\infty }{\underset{t=0}{\sum }}\gamma^{t}I\left(s_{t}=s^{\prime }\right)|s_{0}=s\right],$$ [2]

where $I\left(s_{t}=s^{\prime }\right)=1$ if $s_{t}=s^{\prime }$ and 0 otherwise. Each entry $M^{\pi }\left(s,s^{\prime }\right)$ of the SR estimates the exponentially discounted count of the number of times state $s^{\prime }$ is visited in the future, given that the current state is s, conditioned on the current policy π(a|s). In addition to the SR, the hippocampal system learns a vector of rewards R associated to each state, which is multiplied with the SR to compute state values (Eq. **8**). Crucially, the hippocampal SR algorithm learns aggregate statistics over the relational structure between states, which allows for some of the flexibility of fully MB systems at lower computational cost. Specifically, SR-based systems decouple learning about transition dynamics from learning about reward, which allows for a quick recomputation of value under a new reward distribution.

Arbitration between the two systems was achieved by tracking their reliability in predicting states (HPC) and rewards (DLS) and weighting either systems’ action values by this reliability, following Wan Lee et al. (43). We operationalized this as the average recent reward-prediction error for the MF system and as the average successor state-prediction error for the SR system. These reliability measures were then used to compute the proportion of influence the SR system had on the value function, $P_{SR}$ (see Eq. **18** for details). Although not modeled in detail here, we suggest that this arbitration is supported by the medial prefrontal cortex, following previous theoretical and experimental work (2, 52). Fig. 1 *B*–*D* shows an example of how the arbitrator functions. The agent was trained to find a reward (given with probability 0.8) at the end of a simple linear track, in which each state was uniquely identified by landmarks (Fig. 1*B*). The agent was allowed to explore the environment randomly, so it started with a random-walk SR. Hence, the reliability of the HPC starts out higher than that of the DLS. As the average DLS reward-prediction error goes down, and its reliability catches up with that of HPC, the proportion of HPC influence decreases.

To test the validity of our model, we applied it to spatial and nonspatial decision-making tasks and compared its behavior to that of humans and rodents.

### Hippocampal Lesions and Adapted Water-Maze Navigation.

**Fig. 2.**
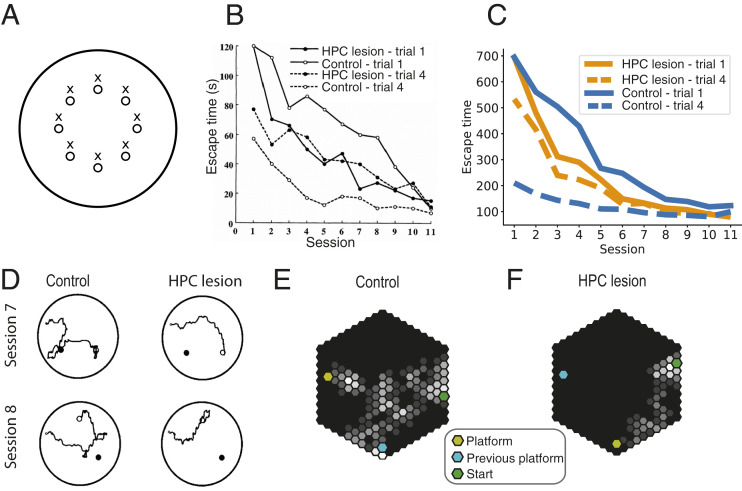
Results and simulations of the experiment are described in ref. 44. Sessions lasted four trials, and platform and landmark were moved at the beginning of each session. (*A*) Possible locations of the hidden platform (o) and the corresponding landmark (x) in each session. (*B*) Escape latency in the water maze for hippocampal lesioned and control animals on trials 1 (solid lines) and 4 (dashed line) of each session. Hippocampal damage impairs intrasession learning, but preserves learning across sessions. Because animals with hippocampal damage follow a response strategy based on egocentric visual input, they perform better on the first trial of each session than control animals. Reprinted from ref. 15. Copyright (2015), with permission from Elsevier. (*C*) Equivalent plot for the full model (blue) and the model without a hippocampal component, relying solely on MF mechanisms. (*D*) Example trajectories from the first trials of sessions 7 and 8. Animals using a hippocampal place strategy tend to wander around the previous platform location (filled circles) before finding the new platform location (open circles) (adapted from ref. 44). (*E* and *F*) Occupancy maps show a similar effect for simulated agents. Control agents (*E*) linger around the previous platform location, whereas agents that cannot use map-based navigation take a more direct path to the new platform location.

An adaptation to the classic Morris water-maze task—in which rodents swim in opaque water to find an invisible platform—involved putting an intramaze landmark into the pool at a fixed offset from the platform and moving both platform and landmark to a different location within the tank at the start of each block of four trials (ref. 44 and Fig. 2*A*). In this version of the task, hippocampally lesioned animals performed *better* than intact animals on the first trial of each session, because intact animals initially lingered at the previous goal location (Fig. 2*B*). However, these animals showed little intrasession learning, while learning across sessions was relatively unimpaired, indicating that they were learning to navigate to the goal location relative to the landmark, since this relationship remained constant across sessions.

In the model, the session-by-session displacement of landmark and platform means that the value function will have to change when using allocentric place-cell features, but not when using egocentric LC features. Hence, when we simulated this task by comparing the performance of the full model to a model with a silenced hippocampal component, our model showed the same effects as in the original experiments (Fig. 2*C*). Fast within-session learning, which relies on the SR’s capacity for quick reevaluation of rewards, was impaired after a hippocampal lesion. Between-session learning, which depends on learning the landmark–platform relations, was unimpaired. Finally, control agents performed worse than hippocampally lesioned agents on the first trial after the platform had been moved, because the value function changed in allocentric, but not egocentric, coordinate frames. An inspection of the occupancy maps (Fig. 2 *D*–*F*) reveals that equivalent errors were made by the agents and by the rats—i.e., lingering at the previous platform location. The hippocampal predictive map guides the agent to the previous platform location because of its allocentric place representation. Only when it reaches that location and the platform is not there does it start unlearning the hippocampal reward representation; Eq. **11**.

Simulating DLS lesions in the task used by Pearce et al. (44) showed the emergence of the opposite pattern to that of HPC lesions: There was little to no learning across sessions for the first trials, while fourth-trial performance was not significantly worse than control performance (*SI Appendix*, Fig. S2*A*). This is consistent with previous findings showing that lesions of the DLS induced a preference for place-guided navigation (53) and that dopamine depletion in the DLS impairs egocentric, but not allocentric, water-maze navigation (54). Our model also accurately captures results from Miyoshi et al. (55), who classified navigation behaviors as cue-guided or place-guided in the cued water-maze task after lesions to both the HPC and the DLS (*SI Appendix*, Fig. S2 *B* and *C*).

These results show that our model captures both landmark-guided and place-memory-guided behavior on the water maze. Furthermore, our model gives a normative perspective on why the animals switch to a landmark-based strategy: Since the striatal system learns about the rewarded location with respect to landmarks, it can use the landmark to navigate directly to the correct location on the first trial of a given session. This gives an advantage to using the striatal system for decision making, which agents learn to exploit. Over the course of multiple sessions, the average prediction error of the striatal system will decrease, causing the reliability-based arbitration mechanism to favor the striatal system, driving lower escape times on first trials of later sessions.

### Animals Switch to a Response Strategy on the Plus Maze.

**Fig. 3.**
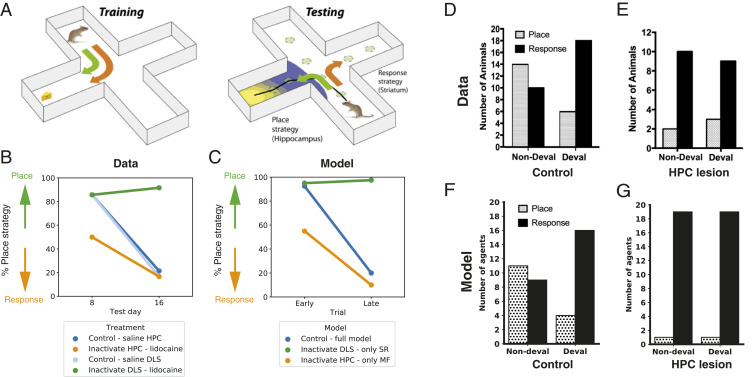
Navigation in the plus maze. (*A*) Experimental setup used by ref. 23. During training, animals were trained to run from the same starting place to a baited goal arm. During probe trials (on day 8 and day 16), the animal started in the opposite arm. If the animal ran to the same allocentric location as during training, this was labeled as a place strategy (green). Taking the same egocentric turn to end up in the opposite goal arm was classified as a response-learning strategy (orange). (*B*) Behavioral data from ref. 23. Control animals (blue) showed a shift to response learning over the course of training. This was prevented by the inactivation of DLS using lidocaine. The inactivation of HPC using lidocaine caused animals to use a response strategy early on. (*C*) Model results recapitulate these findings. (*D* and *E*) Behavioral data from ref. 56 showing probe-trial behavior before and after the outcome was devalued (deval) by prefeeding the animal with the food reward, for control (*D*) and hippocampally lesioned animals (*E*). *D* and *E* are reprinted from ref. 56, which is licensed under CC BY 4.0. (*F* and *G*) Model-simulation results recapitulate these findings.

The distinct roles of the HPC and dorsal striatum have also been investigated by using the place/response learning task (23, 24). In this task, rats were trained to find a food reward on one arm of a plus maze, starting in the same arm every time, while the opposite arm was blocked (Fig. 3). After training, a probe trial was performed, in which the animal started at the opposite end of the maze. If animals take the same egocentric turning direction as before, thus ending up at the opposite goal arm, their strategy is interpreted as response learning (relying on a remembered egocentric turn). If they take the opposite turn to end up in the same goal arm, their strategy is interpreted as flexible place learning (relying on an allocentric representation of space).

Fig. 3 shows the results of the original experiment and our simulations. Early in training, most control rats (injected with saline) used a place strategy, but switched to a response strategy after extensive training. Inactivation of the dorsal striatum with lidocaine prevented this switch. Inactivation of the HPC, by contrast, caused the response strategy to be used more often, even early in training. These results indicate that the dorsal striatum supports response learning, while the HPC supports place learning. We simulated the lidocaine inactivation of HPC and dorsal striatum by partly deactivating the SR and MF components of our model, respectively. Early in training, the control agent showed a preference for actions proposed by the HPC, leading the agent to follow a place strategy. This is because the SR reliability was higher than the MF reliability at the start of training, reflecting the fact that animals have explored the environment without rewards before training. Over the course of training, reward-prediction errors in the striatum decreased, causing the reliability of the MF system to increase, at which point the model switched to the MF strategy because of a bias to use the more computationally efficient system. Inactivation of the dorsal striatal and hippocampal components of the model biases the agent to follow a place or response strategy, respectively.

While the results described above show that the DLS and HPC are involved in egocentric and allocentric navigation, respectively, the navigational strategy alone does not speak to an important aspect of MB learning: flexibility in the face of reward devaluation. In devaluation studies, the value of a reinforcer is decreased by pairing it with an aversive event such as illness or by inducing satiety by prefeeding the animal with the reinforcer (57). Since MF algorithms need to reexperience the state/action leading to the devalued reward to update its value, MF behavior (also referred to as stimulus–response learning) is insensitive to devaluation. MB algorithms, in contrast, can estimate that state/action transitions will lead to a devalued reward without having to reexperience them. This goal-directed, devaluation-sensitive behavior is a hallmark of MB planning (2, 58).

To investigate the relationship between place and response learning on one hand, and goal-directed and stimulus–response learning on the other, we simulated results from Kosaki et al. (56), who studied devaluation on the plus maze. Specifically, they trained rats on the same task as described in Fig. 3*A* (see ref. 59 for a similar study in mice). Subsequently, they devalued the food reinforcer by prefeeding the animals. The results of this devaluation procedure are depicted in Fig. 3*D*. Consistent with the idea that the place strategy is sensitive to the expected value of the outcome, while the response strategy is not, the procedure resulted in a switch from place to response strategies. Furthermore, rats with hippocampal lesions displayed a reliance on the response strategy, regardless of outcome devaluation (Fig. 3*E*), further indicating that the response strategy is insensitive to devaluation. Since sensitivity to reward devaluation is also a property of SR-based learning (60), our model naturally accommodates these results.

### Blocking in Landmark But Not Boundary-Related Navigation.

A signature of learning stimulus–reward associations using reward-prediction errors is the blocking phenomenon (61). Learning one stimulus–reward association hinders learning of a subsequent association between a different stimulus and the same reward because the prediction error becomes small, reducing further weight updates. In humans, spatial blocking has been shown to occur when learning locations relative to discrete landmarks, but not relative to boundaries (27). Furthermore, learning with respect to landmarks corresponds to increased blood-oxygen-level-dependent (BOLD) signal in the dorsal striatum, whereas learning with respect to boundaries corresponds to activity in the posterior HPC (26).

**Fig. 4.**
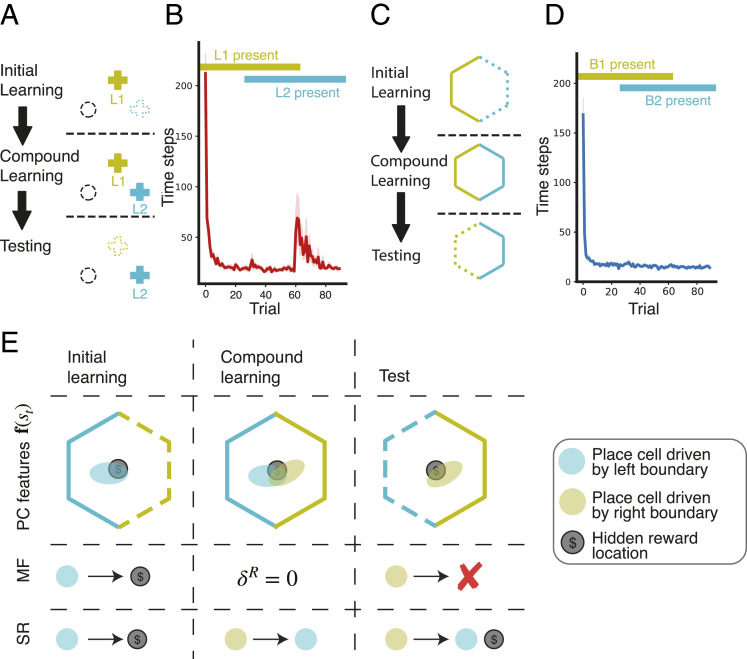
Boundary versus landmark-blocking experiments, similar to ref. 27. (*A*) Landmark blocking experiment. Agents navigate a virtual water maze to find a hidden platform (dashed circle). During initial learning, one landmark is present (L1). During compound learning, a second landmark is added (L2), after which L1 is removed. (*B*) Average time to find the platform per trial. Increased escape times on removal of L1 indicates blocking of learning about platform location relative to L2 by the prior learning relative to L1. (*C*) Boundary-blocking experiment, following *A*, but with two boundaries (solid green and blue lines). (*D*) Average escape time shows no effect of blocking of learning platform location relative to the right boundary (blue) when the left boundary (green) is removed. (*E*) Illustration of the lack of blocking in boundary-related learning under the SR system, in contrast to an MF system.

We aimed to capture these effects by examining the behavior of our agent, following a paradigm similar to ref. 27 (Fig. 4): The agent navigated through an open field to find an unmarked reward location. In order to investigate blocking with respect to boundaries, we explicitly modeled the effect of boundaries on hippocampal place cells, given their dominant role in determining place-cell firing fields (cf. 62 and 63). Rather than learning an SR over a punctate-state representation, the agent learned a matrix of successor features provided by the firing rates of a set of place cells driven by boundary vector cells (BVCs) (64–67).

In the landmark blocking condition (Fig. 4 *A* and *B*), the agent used a landmark to guide navigation. After 10 trials, a second landmark was added, and after 20 trials, the first landmark was removed. Importantly, in this experiment, there were no boundaries, and only one or two landmarks were visible at any time. A single landmark has little effect on place cell firing (63), and, indeed, the presence of a single or two landmarks does not support a reliable place-cell map (64). Therefore, and consistent with BOLD activation results (26), we assume that behavior was controlled by the DLS in this experiment.

As predicted by the TD learning rule, and consistent with the findings of Doeller and Burgess (27), learning about the second landmark was blocked by the prior learning about landmark 1, as evidenced by the drop in performance after its removal.

In the boundary-locking condition (Fig. 4 *C* and *D*), there were no landmarks, meaning that the agent had to rely on its hippocampal system for navigation. The hippocampal system learns a predictive map over boundary-related place-cell activations using successor-prediction errors (SPEs; *SI Appendix*). Prediction-error-based learning like that is susceptible to the blocking effect, and the SR has indeed been used as an explanation for the occurrence of blocking, when learning stimulus–stimulus associations (60). However, when we subjected the agent to a boundary-related blocking paradigm, no blocking occurred (Fig. 4 *C* and *D*).

To understand why this happens, consider the situation in Fig. 4*E*, in which one example place cell was active at the rewarded location, driven by the left boundary. During initial learning, an association between that place cell and the reward was learned. During compound learning, a second boundary drove the activity of another place cell at the rewarded location. In an MF system, the learned value associated to the previous place cell means there was zero prediction error, preventing learning of an association between the second place cell and the reward. In an SR system, however, the agent learns a predictive relationship between the two place cells. Thus, while there is no reward-prediction error, and the reward vector remains unchanged, the newly firing place cell comes to predict the firing of the first place cell (that is associated with reward), mitigating its reduction in firing when the first boundary is removed. This means that, when the first boundary and its associated firing are removed, the agent still predicts reward at the correct location. Thus, consistent with behavioral evidence (26, 27), our model shows no blocking effect during the boundary-related navigation paradigm. This result speaks to the utility of structure learning: The hippocampal SR system learns a multitude of relations, such that its policies are more robust to change in cues and rewards.

### Two-Step Task.

**Fig. 5.**
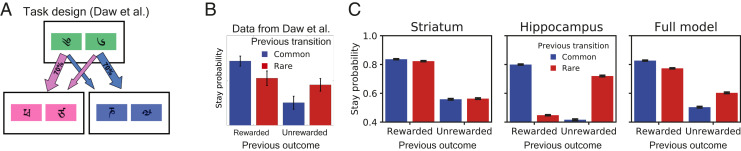
A nonspatial two-step task. (*A*) Task employed by Daw et al. (46). Here, a single start state led probabilistically to one of either two second states, depending on the action chosen and whether by chance a rare (70%) or common (30%) transition was made. (*B*) Data from Daw et al. (46) showing that human performance lies in between MF and MB. *A* and *B* are reprinted from ref. 46, which is licensed under CC BY 3.0. (*C*) Simulation results for the striatal (*Left*), hippocampal (*Center*), and full (*Right*) models.

Outside of the spatial domain, the distinction between MF and MB RL has been heavily investigated by using sequential decision tasks. Here, we describe how our model solves a cognitive decision task of this type—the task of Daw et al. (46) (Fig. 5*A*).

In the two-step decision task designed by Daw et al. (46), human participants were shown a pair of symbols and asked to choose one (Fig. 5*A*). Left or right choices lead to different corresponding second-stage states with high probability (common transitions), but there was a small probability (rare transitions) that the agent transitions to the opposite state. For example, in Fig. 5*A*, the left icon in the first (green) state usually leads to the choice in the pink state (common transition), but occasionally leads to the choice in the blue state (rare transition). During the second stage, participants made another left-or-right choice, resulting in either receiving a reward or not, before starting the next trial. Each of the four outcomes was associated with a reward probability that varied over time as a Gaussian random walk limited between 0.25 and 0.75.

The rewards received or not received on a given trial modify the participants’ value estimates for the different actions taken during the two stages, but different RL strategies lead to different behaviors on the next trial. MF learners increased the likelihood of repeating their first-stage action following a reward, regardless of whether a common or rare transition was made. In contrast, MB learners used knowledge of the task’s transition structure, such that rewards obtained after a rare transition lead to the opposite choice on the next trial (to maximize the likelihood of reaching the same second state). The key finding of Daw et al. (46) was that human choices reflect both MB and MF influences (Fig. 5*B*).

Our model recapitulates these findings and suggests the HPC could support MB choice in this task, as well as another two-step decision task with deterministic transitions (*SI Appendix*, Fig. S3 and ref. 45). The model DLS, implementing an MF RL system, increased stay probability after rewards, regardless of whether a rare or common transition was made (Fig. 5*C*). In contrast, the HPC uses the SR to generalize value over the graph. When a goal state is reached and a reward is obtained, value is generalized over the graph, according to the degree to which states predict each other. Therefore, on the next trial, the actions were taken that will most likely lead to the recent goal state. Separating transition dynamics from reward estimates thus recapitulates true MB behavior. Combining the two systems results in behavior that is similar to that of human participants in this task.

It has been shown that other, simpler models than pure MB systems can look like MB agents on the two-step task (68). Here, we show that the SR can mimic MB behavior. Because the transition structure is unchanging, caching future state predictions is sufficient for flexible behavior.

### Relationship Between Spatial and Two-Step Tasks.

**Fig. 6.**
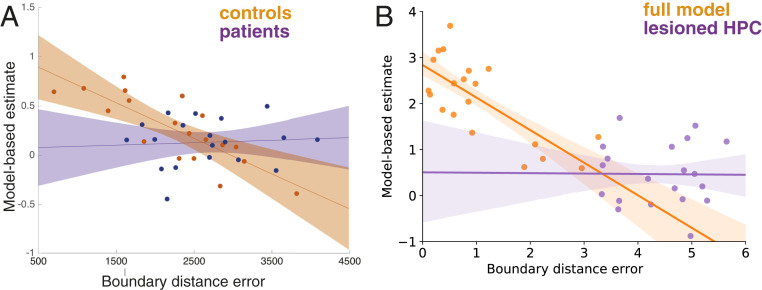
Relationship between MB planning and allocentric spatial memory. Error bars indicate 80% CIs of the regression in both panels. (*A*) Data from healthy control participants and anterior temporal lobectomy patients, from ref. 29. Allocentric place memory is reflected by responses close to the boundary-predicted location after the landmark has moved (i.e., smaller boundary-distance errors). Dots indicate MB estimates for individual participants, calculated from a mixed-effects logistic regression. Reprinted from ref. 29. Copyright (2019), with permission from Elsevier. (*B*) Simulation data for the full model and agents for which the HPC component was turned off. Here, allocentric place memory is reflected by the average distance between the previous platform location and the location of the maximum of the agent’s value function at the start of the next session. Dots represent estimates for individual agents, estimated by a mixed-effects logistic regression.

A central principle of our model is that MB reasoning and allocentric navigation strategies both rely on the same hippocampal structures. The most direct evidence for this comes from Vikbladh et al. (29), in which both healthy participants and patients with hippocampal damage performed the two-step planning task (46), as well as a landmark versus boundary spatial memory task (26). This allowed the authors to show that, in healthy participants, the degree of MB planning on the sequential decision task correlated with the contribution of allocentric, boundary-driven place memory on the spatial task (reflected in smaller errors from the location predicted by the boundary; Fig. 6*A*). Notably, this correlation cannot be accounted for by variation in general intelligence (intelligence quotient). In patients with hippocampal damage, however, this relationship was significantly reduced.

To test for this effect in our model, we sampled a set of 20 agents with different values for the parameters governing the hippocampal–striatal tradeoff, as well as 20 agents with a partially lesioned hippocampal component (*SI Appendix*). Each agent performed the two-step decision task (46) and the water-maze task of Pearce et al. (44), depicted in Fig. 2. MB planning was quantified as the interaction between effects of reward and transition type in the previous trial on staying with the same action or switching in the next trial (*SI Appendix* and cf. refs. 29 and 46). We quantified the degree of allocentric place memory as the average distance between the previous platform location and the location of the maximum of the agent’s value function at the start of the next session. This is akin to the boundary distance error employed by ref. 29. We found a significant correlation (z=1.89,p<0.001) between model based and allocentric planning (Fig. 6*B*). Agents with hippocampal lesions did not show a significant correlation (z=−0.02,p=0.97), and the difference between these correlation coefficients was significant (z=5.44,p<0.001), recapitulating the result found by Vikbladh et al. (29).

## Discussion

We presented a model of hippocampal and dorsolateral striatal contributions to learning across both spatial navigation and nonspatial decision making. Our simulations support the view that the HPC serves both allocentric place learning and flexible decision making by supplying a predictive map of the underlying structure of the task or environment, whereas the DLS underlies MF learning based on (egocentric) sensory features and actions and that these systems combine weighted by their relative reliability in predicting outcomes.

The involvement of the HPC in abstract nonspatial tasks raises questions about its role throughout evolution. Did the system evolve initially in the spatial domain, but become recruited more generally (14), or was spatial decision making always part of a more general ability (69)? The role of the HPC in MB decision making is much debated. On one hand, lesions of the HPC have not affected hallmarks of MB planning, such as outcome devaluation in lever-pressing tasks (32, 33), although a recent study showed that HPC is involved in devaluation sensitivity of lever pressing immediately after acquisition (when pressing is context-dependent; ref. 70). On the other hand, hippocampal lesions led to a loss of devaluation-sensitivity on the plus maze (Fig. 3 and ref. 56) and impair MB behavior on the two-step task (Fig. 5 and refs. 28 and 29). One crucial difference between the lever-pressing tasks and the tasks simulated here is that the lever-pressing tasks required only one action–outcome association, whereas solving the two-step task and many spatial tasks require chaining multiple action–outcome associations together. Perhaps then, as suggested by Miller et al. (28), the HPC is specifically required when planning requires linking actions to outcomes over multiple steps. By storing temporal abstractions of future states separately from a representation of reward, the SR is particularly well suited for this task of rapidly propagating novel reward information to distant states. That property of the SR has previously inspired models of temporal context memory (71) and might also relate to the role of relational memory tasks more broadly, as they require chaining multiple stimulus–stimulus associations together (37, 39). In line with this role, our simulations showed the hippocampal SR as driving a correlation between spatial-memory performance and MB behavior (Fig. 6 and ref. 29).

Consistent with our model, dorsal striatal neurons showed a great degree of spatial coding in spatial tasks (72), but not in tasks where reward locations were explicitly dissociated from space (73) or where multiple locations were equivalently associated with rewards (74). Indeed, dorsal striatum selectively represents those task aspects, which computational accounts suggest are important for gradual, MF learning (72).

We specifically associate our striatal model with the DLS. Lesion and inactivation studies have shown that the dorsal striatum is functionally very heterogeneous (75). Lesions of the dorsomedial striatum (DMS) result in a switch to response strategies on the plus maze (76) and to cue-based responding in the water maze, while the DLS underlies response learning (77). Furthermore, the DMS has been implicated in learning action–outcome contingencies outside the spatial domain (21, 75). Anatomical connectivity supports this functional dissociation in the dorsal striatum (53, 75). Whereas the DLS receives inputs mostly from sensorimotor cortex and dopaminergic input from the substantia nigra, the DMS receives input from several mesocortical and allocortical areas including the HPC. Indeed, cells encoding route and heading direction have been found in the DMS (78, 79). It is, therefore, likely that the dorsal HPC and the DMS are part of a single circuit involved in flexible goal-directed decision making, whereby the HPC provides map-based information, and the DMS is involved in action selection.

Our work follows several models of spatial decision making by hippocampal and striatal systems (15, 48, 49, 80, 81). Dollé and colleagues (48, 49) used a similar hippocampo-striatal model to explain behavior on the adapted water-maze task (44), presented in Fig. 2. Our model differs in two important ways. Firstly, in their model, place cells connected to “graph cells” that formed an explicit topological graph of the spatial environment, used to explicitly plan a path to the goal. In the present model, by contrast, the topological structure of the environment is implied in the predictive SR, following a theoretical proposal by Stachenfeld et al. (51) and neuroimaging (40, 41) and behavioral findings (82). Thus, our agent mimicked true MB behavior (explicit graph search) by using an intermediate SR-based strategy. Secondly, their model used another expert network that learned whether to take striatal or hippocampal outputs using TD learning. In contrast, our model arbitrates between systems based on their reliability. This arbitration mechanism predicts that on trials with high reward-prediction error, control should shift away from the MF system. In contrast, a low predictability of state transitions leads to higher average errors in the SR system and should, therefore, lead to a higher degree of MF control. Evidence for this comes from Wan Lee et al. (43), who, furthermore, showed that the prefrontal cortex encodes neural correlates of arbitration based on reliability.

As noted above, the hippocampal results we simulated are also consistent with a fully MB system, which is strictly more flexible. An interesting question is how to disambiguate between animals using an MB strategy versus the SR. One weakness of the temporal-difference SR model used here is that it cannot respond flexibly when the transition structure changes. Momennejad et al. (83) have shown that humans are better at revaluating when the reward function changes than when the transition structure changes, consistent with use of an SR. In addition, hippocampal replay has been suggested to perform off-line updates of the hippocampal predictive map to incorporate these kinds of transition changes (84, 85). As an alternative, tracking input covariances and using these for updating the SR allow it to solve certain kinds of transition-revaluation problems without requiring forward simulation (86). A second weakness of the SR, compared to MB systems, is that the SR is policy-dependent. This means that the SR corresponding to an optimal policy for one reward setting is of limited use for problems with a different reward function (87). Piray and Daw (88) have recently proposed that the hippocampal system might resolve this latter weakness using a *default representation*, corresponding to a default policy. Alternatively, the HPC might represent a set of multiple distinct SR maps corresponding to different policies (89). Taken together, these two failure modes of the SR provide interesting avenues for experiments probing animals’ behavioral strategies and for theoretical work on computational tradeoffs between these strategies.

In addition to the HPC, the orbitofrontal cortex (OFC) has been hypothesized to be important for representing states in RL problems. Wilson, Niv, and colleagues (90) introduced a model in which OFC plays a critical role in identifying states that are perceptually similar. This corresponds to data showing that OFC is specifically necessary for decision making in partially observable environments (91). Evidence for this theory comes from human functional MRI research showing that unobservable task states can be decoded from OFC and that this relates to task performance (92). This proposed role of the OFC is distinct from, and possibly complementary to, our proposed role for the HPC. In our model, the HPC encodes a predictive map based on observable features that can be used for rapid, flexible decision making. The OFC, on the other hand, is crucial for a general state representation that can be used for downstream MB or MF processes. Whether and how the OFC and the HPC can interact to allow SR learning in partially observable environments is an interesting avenue for further research (see also ref. 93).

Our explanation for the absence of boundary-related blocking (Fig. 4) relies on BVC inputs to hippocampal place cells. BVCs can respond to intramaze landmarks as well as to boundaries (although, in contrast to DLS LCs, BVCs fire irrespective of object identity; ref. 67). This means that a sufficient number of landmarks could drive a reliable place-cell representation of space, allowing hippocampal control and the prevention of blocking. However, in the experiments simulated here, there were only one or two landmarks present. Single landmarks have little influence on firing relative to extended boundaries (63), consistent with the BVC model. Because BVCs fire proportionally to the angle subtended by the stimulus (94), place cells do not provide a reliable representation of space when there is only a single landmark (64). Thus, we predict that the addition of greater numbers of landmarks should allow construction of a reliable place-cell map, thereby leading to increased hippocampal influence and a reduction of blocking effects.

Our model reflects the assumption, driven by our knowledge of the neural representations, that in spatial tasks, the hippocampal SR system uses allocentric representations, while the MF system uses egocentric representations. This allowed us to fit the behavioral data well and raised the question of why the goal-directed system is allocentric, while the stimulus–response system is egocentric? Perhaps an answer lies in the time scale of learning: The allocentric layout of a large environment is stable, irrespective of your changes in location or direction, making it suitable for learning long-term relationships between stimuli. Consistent with this idea, “slow feature analysis” produces grid and place-cell representations from visual inputs because they vary slowly (95). On the other hand, egocentric representations are more suited to mapping sensory inputs to physical actions, both of which are specified egocentrically.

In conclusion, dorsal HPC and DLS support qualitatively different strategies for learning about reward in spatial as well as nonspatial contexts, as captured by the model presented here. The fact that the same model explains behavior in both types of tasks implies that the hippocampal–striatal system is a general-purpose learning device that adaptively combines MB and MF mechanisms.

## Materials and Methods

### Hippocampal and Striatal Systems for Decision Making.

Our model combines a hippocampal RL module based on the SR with a striatal model based on MF value learning (Fig. 1*A*). It arbitrates between these modules based on their relative reliability, which can be computed by using the average of recent prediction errors. Model details are outlined below.

### Dorsal Striatal System.

The DLS module was implemented as an MF RL system that learned direct associations between sensory stimuli and actions. Striatal neurons coded for the value of each action, where actions were expressed as egocentric-heading directions in the spatial-navigation tasks and left or right button presses in the nonspatial tasks. Sensory input was coded by a set of egocentric landmark vector cells coding for the presence or absence of a landmark in a particular egocentric direction, at a particular distance from the landmark to the agent, analogous to the egocentric BVCs recently reported (96). Specifically, the activation of each LC was modeled as a bivariate Gaussian in a space defined by the egocentric angle θ and distance d of the landmark to the agent:

$$f^{LC}\left(d,\theta \right)\propto N\left(\left[d,\theta \right];\left[d*,\theta *\right],\Sigma \right),$$ [3]

where d* and θ* are the preferred distance and orientation of the LC, respectively, and $\Sigma =diag\left(\left[\sigma_{d},\sigma_{\theta }\right]\right)$ is the covariance matrix with the tuning width and length of the receptive field on the diagonal entries. We assumed that LCs are sensitive to the identity of the landmark, meaning that a different set of LCs will respond to a different landmark in our model. An example egocentric LC is shown in *SI Appendix*, Fig. S1. In the nonspatial tasks, states were encoded as “one-hot” vectors containing ones for their state indexes, reflecting the fact that states were uniquely identifiable as different images.

LCs in the sensory layer project to neurons in the dorsal striatum in an all-to-all connected way:

$$x_{a}^{\text{DLS}}=Q_{\text{DLS}}\left(s,a\right)=\overset{N}{\underset{i=1}{\sum }}w_{i,a}f_{i}^{\text{LC}}\left(s\right),$$ [4]

where $f_{i}^{\text{DLS}}$ is the activity of LC i, $x_{a}^{\text{DLS}}$ is the firing rate of the dorsolateral striatal neuron corresponding to striatal estimated value $Q^{\text{DLS}}$ of action a given state s, N is the total number of sensory neurons, $u_{i}^{\text{LC}}$ is the firing rate of LC i, and $w_{i,a}$ is the weight from sensory neuron i to striatal neuron a.

Learning in the striatal network is mediated by a Q-learning rule (50). This allows the model to compute a TD reward-prediction error $\delta_{t}^{r}$:

$$\delta_{t}^{r}=r_{t+1}+\gamma \underset{a^{\prime }}{\max}Q_{\text{DLS}}\left(s_{t+1},a^{\prime }\right)-Q_{\text{DLS}}\left(s_{t},a_{t}\right),$$ [5]

where $r_{t+1}$ is the reward received at time t+1. This prediction error is then used to update the weights:

$$\Delta w_{i,a}=\alpha_{Q}\delta_{t}^{r}e_{i,a},$$ [6]

with learning rate $\alpha_{Q}$ and eligibility trace $e_{i,a}$, which tracks which weights are eligible for updating based on recent activity. Every time step, the eligibility trace is updated according to the following rule:

$$e_{i,a}\left(t+1\right)=f_{i}^{LC}x_{a}^{DLS}+\lambda e_{i,a}\left(t\right),$$ [7]

where λ is the trace-decay parameter, controlling for how long synapses stay eligible for updating. Eligibility traces enable faster learning by making it possible to update weights that were active in the recent past instead of only the very last time step (1).

### Hippocampal System.

The hippocampal place-cell system was modeled as encoding the SR, following work by Stachenfeld et al. (51). The SR is a predictive representation employed in machine learning (11, 13, 97, 98), containing the discounted future occupancy of each state $s^{\prime }$ from current state s (Eq. **2**). In the hippocampal SR model, a row of the SR—i.e., $M^{\pi }\left(s,:\right)$—constitutes the current population activity vector—i.e., the activity of every place cell in the current state. A column of $M^{\pi }$ contains the activity of a single place cell in all possible locations (states)—i.e., a rate map (*SI Appendix*, Fig. S1). In addition to the SR matrix, the agent will learn a vector with the expected reward R(s) for each states. The agent combines these to compute state value:

$$V_{\text{HPC}}^{\pi }\left(s\right)=\underset{s^{\prime }}{\sum }M\left(s,s^{\prime }\right)R\left(s^{\prime }\right).$$ [8]

The factorization of value into the SR and reward confers more flexible behavior because if one term changes, it can be relearned, while the other term remains intact (11). The agent used one-step lookahead to compute the value of each action Q(s,a), combining direct reward and the next state’s value:

$$Q_{\text{HPC}}\left(s_{t},a_{t}\right)=r\left(s_{t}\right)+\gamma E_{s_{t+1}|s_{t},a_{t}}\left[V_{\text{HPC}}\left(s_{t+1}\right)\right].$$ [9]

The SR satisfies a Bellman equation, meaning that any RL method can be used to learn the SR. Here, learning was achieved by using a TD update:

$$\Delta \hat{M}\left(s_{t},s^{\prime }\right)=\alpha_{M}\delta_{t}^{M}\left(s^{\prime }\right),$$ [10]

where $\delta_{t}^{M}\left(s^{\prime }\right)=\left[I\left(s_{t}=s^{\prime }\right)+\gamma \hat{M}\left(s_{t+1},s^{\prime }\right)-\hat{M}\left(s_{t},s^{\prime }\right)\right]$ is a TD SPE pertaining to state $s^{\prime }$ and $\alpha_{M}$ is a learning rate. For the spatial-navigation studies modeled in this paper, animals were allowed to freely explore the environment without any reward before starting the task (23, 44). Hence, for these tasks, the SR was initialized as the SR associated to a random-walk policy $M^{RW}$ over a uniform spatial discretization of the environment. This was not the case for the task graphs of the two-step decision tasks (45). Therefore, in these tasks, we initialized the SR as the identity matrix I, encoding no other knowledge than the fact that every state predicts itself. Finally, the reward vector $\hat{R}$ was learned by using a simple delta rule:

$$\Delta \hat{R}\left(s_{t}\right)=\alpha_{R}\left(r_{t}-\hat{R}\left(s_{t}\right)\right).$$ [11]

Although the SR is often introduced as above (in terms of discrete state counts), accurately estimating the SR for every state is infeasible in very large state spaces. This is known as the *curse of dimensionality*, and it necessitates the use of function approximation (1). The agent observes states through a vector of features f(s), which, if chosen rightly, will be of much smaller dimension than the number of states, allowing the agent to generalize to states that are nearby in feature space. The feature-based SR [also referred to as Successor Features (13)], rather than encoding the discounted number of state visits, encodes the expected discounted future activity of each feature:

$$\psi^{\pi }\left(s\right)=E_{\pi }\left[\overset{\infty }{\underset{t=0}{\sum }}\gamma^{t}f\left(s_{t}\right)|s_{0}=s\right].$$ [12]

As in the tabular case, the feature-based SR can be used to compute value when multiplied with a vector of reward expectations per feature, u: $V^{\pi }\left(s\right)=\psi^{\pi }{\left(s\right)}^{T}u$. In the case of linear-function approximation, these Successor Features ψ in Eq. **12** are approximated by a linear function of the features f:

$$\hat{\psi }\left(s\right)=W^{T}f\left(s\right),$$ [13]

where W is a weight matrix which parameterizes the approximation. Intuitively, W encodes how much each feature predicts every other feature. As in the tabular case, TD learning can be used to update the SR weights (*SI Appendix*). Thus, at every state s (corresponding to a location) in the environment, the agent observed a population vector f(s) of BVC-driven place cells. It then computed its estimated Successor Features ψ using its current estimate of weights W and Eq. **13**, which encode the discounted sum of future population firing-rate vectors f of the input place cells. In terms of circuitry, W might correspond to the Schaffer collaterals projecting from CA3 to CA1 neurons, corresponding to f and ψ, respectively.

In the context of HPC, the feature-based SR allows us to represent states as population vectors of place cells with overlapping firing fields (the features), rather than having a one-to-one correspondence between place cells and states. Then, we are free to model the dependence of the place cell firing on specific environmental features (boundaries). This dependence has been extensively characterized by computational models of BVCs (64, 65, 99–101), which were shown to exist in the subiculum (66). Accordingly, we modeled a set of hippocampal place cells, whose activity $f_{i}\left(s_{t}\right)$ was the thresholded sum of a set of BVC inputs (see ref. 64 for details on how BVC and place-cell maps were calculated).

Crucially, modeling place cells as driven by BVCs allows us to explain the puzzling experimental finding by Doeller and Burgess (27) that learning to navigate to a location relative to a landmark, but not relative to a boundary, is sensitive to the blocking effect (61). In an accompanying neuroimaging paper, the authors showed that landmark learning was associated to BOLD activity in the dorsal striatum, whereas boundary-related navigation was associated to activity in the HPC (26).

### Arbitration Process.

The agent has access to both its MF DLS component and its hippocampal component employing the SR. Both systems estimate the same value function, but might make different types of errors, and the agent has to arbitrate between them.

Rational arbitration should reflect the relative uncertainty (2), requiring the posterior distribution over values, rather than just the values themselves. Here, we used a convenient proxy for uncertainty, introduced by Wan Lee et al. (43)—namely, the recent average of prediction errors: the reward-prediction error for the MF component and the SPE for the SR component. If the SPE is low, this means that the SR system has a good estimate of the world. Similarly, if reward-prediction errors are low, this means the MF system has a reliable estimate of the value function. The reliability can be tracked by using a Pearce–Hall-like update rule (102), computing the recent average of absolute prediction errors Ω:

ΔΩ=η(|δ|−Ω), [14]

where |δ| is the absolute reward-prediction error and η is a learning rate. The reliability is defined as:

$$\chi =\left(\delta_{MAX}-\Omega \right)/\delta_{MAX},$$ [15]

with $\delta_{MAX}$ being the upper bound of the prediction error, which was set to one. Since in our model both systems are trained by a prediction error, we can apply this to both the MF and SR systems. Following Wan Lee et al. (43), we used the reliability measure for arbitration. These authors computed transition rates α and β for transitioning from MF to MB states, and vice versa, as follows. Here, we used the same terms, but for transitions between MF and SR. These transition rates are functions of the reliability of the respective systems:

$$\alpha \left(\chi_{MF}\right)=\frac{A_{\alpha }}{1+\exp\left(B_{\alpha }\chi_{MF}\right)},$$ [16]

$$\beta \left(\chi_{SR}\right)=\frac{A_{\beta }}{1+\exp\left(B_{\beta }\chi_{SR}\right)},$$ [17]

where the A and B parameters in both equations determine the transition rate and the steepness of these curves, respectively. These parameters were fitted to behavioral data by Wan Lee et al. (43), and we matched their parameter values (*SI Appendix*, Table S1). At each time step, the rate of change of the proportion of influence of the SR system $P_{SR}$ was computed by using the following differential equation, generating a push–pull mechanism between HPC and DLS influence over behavior:

$$\frac{dP_{SR}}{dt}=\alpha \left(\chi_{MF}\right)\left(1-P_{SR}\right)-\beta \left(\chi_{SR}\right)P_{SR}.$$ [18]

Note that, consistent with behavioral data from human subjects (43), this arbitration mechanism resulted in a weighted influence of both systems in the final value estimates (Fig. 1), rather than a discrete choice. Note that the arbitrator combines the action values, not the actions. Thus, the agent will not end up with a midway action when the two systems encode different preferences. Lesions or partial inactivations of either the DLS or the HPC were achieved by setting limits on $P_{SR}$ (see *SI Appendix* for more details).

### Code Availability.

The results were generated by using code written in Python. Code is available on ModelDB (accession no. 266836) (103).

## Supplementary Material

Supplementary File
