# Supplementary material for: A general model of hippocampal and dorsal striatal learning and decision making
Source: Proc Natl Acad Sci U S A. 2020 Nov 23;117(49):31427–37. doi: 10.1073/pnas.2007981117 (PMC7733794; doi:10.1073/pnas.2007981117)
Supplement: Supplementary File [file pnas.2007981117.sapp.pdf]

# Supplementary Information for

## A general model of hippocampal and dorsal striatal learning and decision making

**Jesse P. Geerts, Fabian Chersi, Kimberly L. Stachenfeld & Neil Burgess**

**Neil Burgess**
**E-mail: n.burgess@ucl.ac.uk**

### This PDF file includes:

Supplementary text
Figs. S1 to S3
Table S1
SI References

## Supporting Information Text

We describe arbitration in our model in more detail. We then describe task-specific adaptations that were made to the model, and some additional experiments.

## Arbitration between hippocampal and striatal systems

We implemented arbitration between the hippocampal and dorsal striatal systems in our model using a rule introduced by Wan Lee et al. (1). These authors suggested that arbitration between model-based and model-free systems was done based on a *reliability* signal. They also used fMRI to show that inferior lateral prefontal and frontopolar cortex encode such reliability signals, as well as the output of a comparison between these signals. Furthermore, they showed evidence that the connectivity between these regions and model-free value areas is negatively modulated by the degree of model-based control.

Here, we applied their method to arbitration between a hippocampal system based on the Successor Representation and a striatal system based on model-free learning. The idea is that the reliability of both systems is tracked by computing the recent average of prediction errors of both systems. The Pearce-Hall update rule for tracking average prediction error is:

$$\Delta\Omega = \eta(|\delta| - \Omega) \tag{1}$$

where $|\delta|$ is the absolute RPE and $\eta$ is a learning rate. The reliability is defined as:

$$\chi = (\delta_{MAX} - \Omega)/\delta_{MAX} \tag{2}$$

with $\delta_{MAX}$ being the upper bound of the prediction error, which was set to 1. After each episode, the reliability of each system was updated using the following rule:

$$\Delta\chi = \eta\left[\left(1 - \frac{|\delta|}{\delta_{MAX}}\right) - \chi\right] \tag{3}$$

This measure goes to zero as the average prediction error increases ($\Omega \to \delta_{MAX}$), and goes to one as the average prediction error decreases ($\Omega \to 0$).

Following Wan Lee et al. (1), we can use the reliability measure for arbitration. These authors computed transition rates $\alpha$ and $\beta$ for transitioning from MF to MB states and vice versa as follows. Here we use the same terms but for transitions between MF and SR. These transition rates are functions of the reliability of the respective systems:

$$\alpha(\chi_{MF}) = \frac{A_\alpha}{1 + \exp(B_\alpha \chi_{MF})} \tag{4}$$

$$\beta(\chi_{SR}) = \frac{A_\beta}{1 + \exp(B_\beta \chi_{SR})} \tag{5}$$

where the A and B parameters in both equations determine transition rate and the steepness of these curves, respectively. These parameters were fitted to behavioural data by Wan Lee et al. (1) and we matched their parameter values (see Table S1).

At each time step, the rate of changes of the probability of choosing the SR system $P_{SR}$ was computed using the following differential equation:

$$\frac{dP_{SR}}{dt} = \alpha(\chi_{MF})(1 - P_{SR}) - \beta(\chi_{SR})P_{SR} \tag{6}$$

Although not explored here (but see 1), this means that there is a certain "stickiness" to the model: if the model is currently choosing MF actions, it will take some time to move weight to the MB system.

Following Wan Lee et al. (1), state action value estimates were given by a weighted average of the two model components:

$$Q(s,a) = P_{SR}Q_{HPC}(s,a) + (1 - P_{SR})Q_{DLS}(s,a) \tag{7}$$

Thus, the degree to which a system contributes to the value estimate is influenced by its reliability. Given these full-model state-action values, the agent chose actions following a softmax policy:

$$\pi(a|s) = \frac{e^{\tau^{-1}Q(s,a)}}{\sum_{a'} e^{\tau^{-1}Q(s,a')}} \tag{8}$$

where $\tau^{-1}$ is an inverse temperature parameter which sets the balance between exploration and exploitation. The higher the inverse temperature, the more the agent chooses higher-valued actions.

## Task-specific adaptations

Although the general model architecture remained the same throughout all simulations, different adaptations were made to the model described above such that it could be used in the differen state spaces defined by the tasks.

**Plus maze.** For the Plus Maze task described in Fig.3, landmark cells were tuned to the ends of the maze. We assumed that the landmark cells could not distinguish between the two ends of the maze such that, from the point of view of the striatal system, probe trials and training trials looked the same.

**Blocking.** For the blocking simulations (Fig.4), we adapted the hippocampal controller (that worked with a tabular state representation as input) to incorporate the effects of boundaries on place cell firing. To that end, we defined the hippocampal SR system using linear function approximation. The agent observes states through a vector of features $\mathbf{f}(s)$ which, if chosen rightly, will be of much smaller dimension than the number of states, allowing the agent to generalise to states that are nearby in feature space. The feature-based SR (2) encodes the expected discounted future activity of each feature:

$$\psi^{\pi}(s) = \mathbb{E}_{\pi}\left[\sum_{t=0}^{\infty} \gamma^t \mathbf{f}(s_t) | s_0 = s\right] \qquad [9]$$

As in the tabular case, the feature-based SR can be used to compute value when multiplied with a vector of reward expectations per feature, $\mathbf{u}$: $V^{\pi}(s) = \psi^{\pi}(s)^T \mathbf{u}$. In the case of linear function approximation, these Successor Features $\psi$ in Equation 9 are approximated by a linear function of the features $\mathbf{f}$:

$$\hat{\psi}(s) = W^T \mathbf{f}(s), \qquad [10]$$

where $W$ is a weight matrix which parameterises the approximation.

In the context of hippocampus, the feature-based SR allows us to represent states as population vectors of place cells with overlapping firing fields (the features), rather than having a one-to-one correspondence between place cells and states. Then we are free to model the dependence of the place cell firing on specific environmental features (boundaries). This dependence has been extensively characterised by computational models of boundary vector cells (BVCs) (3–7), which were shown to exist in the subiculum (8). Accordingly, we modelled a set of hippocampal place cells whose activity $\mathbf{f}_i(s_t)$ was the thresholded sum of a set of BVC inputs (see 5, for details on how BVC and place cell maps were calculated).

Thus, at every state $s$ (corresponding to a location) in the environment, the agent observed a population vector $\mathbf{f}(s)$ of BVC-driven place cells (see Fig.S1 for an example). It then computed its estimated Successor Features $\psi$ using its current estimate of weights $W$ and Equation 10, which encode the discounted sum of future population firing rate vectors $\mathbf{f}$ of the input place cells. In terms of circuitry, $W$ might correspond to the Schaffer collaterals projecting from CA3 to CA1 neurons, corresponding to $\mathbf{f}$ and $\psi$, respectively.

As in the tabular case, temporal difference learning can be used to update the SR weights:

$$\Delta W = \alpha \left[\mathbf{f}(s_t) + \gamma\psi(s_{t+1}) - \psi(s_t)\right] \mathbf{f}(s_t)^T \qquad [11]$$

Note that the algorithm has not changed with respect to the one-hot state encoding mentioned earlier – it is easy to see that the function approximation version reduces to the tabular case when $\mathbf{f}$ is a one-hot vector. The reward expectation vector $\mathbf{u}$ was updated using a simple delta rule:

$$\Delta\hat{\mathbf{u}} = \alpha \left(r_t - \hat{\mathbf{u}}^T \mathbf{f}\left(s_t\right)\right) \mathbf{f}\left(s_t\right) \qquad [12]$$

**Two-step tasks.** For the non-spatial two-step tasks (Fig.5 and Fig.S3), the DLS cells were assumed to provide a one-hot representation of the task states. While this is significantly different from the landmark cell representation used in the spatial navigation studies, this representation reflected the fact that states were uniquely identifiable as different images. Furthermore, this is consistent with experimental evidence showing that dorsal striatum represents reward-predictive cues (9).

**Hippocampal damage in the two-step and spatial tasks.** In order to mimic the individual differences between participants found by Vikbladh et al. (10), we sampled 20 different agents with varying values for the parameters governing the transition from SR to MF and vice versa (see Equations 4 and 5). Specifically, we sampled $A_{\alpha}$ values (steepness of the transition from MF to SR) uniformly between .5 and 5, and $A_{\beta}$ (steepness of the transition from SR to MF) values uniformly between 2 and .5. In addition to the 20 "full agents", we sampled 20 agents for which the hippocampal component was partially inactivated by setting a maximum to the $P_{SR}$. To mimic variability in the size of the lesion that was present in the dataset of Vikbladh et al. (10), we sampled max $P_{SR}$ values from a uniform distribution between 0 and 0.35.

## Quantification and statistical analysis

To investigate the relationship between the agents' spatial navigation and non-spatial decision making strategies, we quantified the agents' degree of MB planning, as well as their degree of using an allocentric strategy, and computed their correlation.

For quantifying MB planning, we followed earlier studies (10, 11) and analysed the agents' choices using a mixed-effects logistic regression (estimated using the *statsmodels* Python package, (12)). For each trial, the dependent variable (stay with the same first-level action or switch) was explained in terms of whether there was a reward on the previous trial, whether the previous transition was of the rare or common type, and the interaction between these factors. The logic of the two-step task is that an MB learner will stay with the same action if it was rewarded after a common transition, but will be more likely to switch if it gets rewarded after a rare transition. Thus, the degree of MB planning can be quantified as the interaction between previous reward and trial type.

For quantifying the degree of allocentric place memory, we computed the average distance between the previous platform location and the location of the maximum of the agent's value function at the start of the next session. This is akin to the boundary distande error employed by (13).

After computing the correlation between allocentric place memory and MB planning for both the "healthy" and "lesioned" groups of agents, we asked whether the two correlation coefficients were significantly different from each other by applying the Fisher z-transform (14) to the coefficients, and testing whether the difference between the transformed coefficients was significantly different from zero.

For the cued water maze task described in Fig.S2, the differences among the groups in relation to the number of agents that chose a place or a cue strategy were analysed by the Fisher exact test as implemented in R (15).

## Additional tasks

**Cue versus place Water Maze.** In addition to the hippocampal lesion described in Fig.2, we simulated a DLS lesion in the task used by Pearce et al. (16). Fig.S2A shows the simulation results: there is little to no learning across sessions for the first trials of each session, indicating impaired acquisition of the landmark-platform association. Fourth-trial performance is not significantly worse than control performance, which is a sign of intact place learning as agents still learn during a session in which the platform has a fixed location. This is consistent with a previous finding showing that dopamine depletion in the DLS impairs egocentric but not allocentric Water Maze navigation (17). Fig.S2B shows results from a study by Myoshi et al. (18) that investigated the effects of bilateral lesions of the hippocampus, DLS or both in a cue on a probe test in the Water Maze. Animals were trained to swim to a given location in the Water Maze, that was indicated by the presence of a landmark. Then, during a probe trial, the landmark was placed elsewhere in the maze, and the animals' behaviour wass classified as cue-guided if the animal swam directly to the cued platform, as place-guided if it swam directly to the place the hidden platform was the day before, or as thigmotaxic if the animals swam around the edge of the pool. This dual-solution probe trial is akin to the first trial of each session in Pearce et al. (16). Fig.S2C shows that our simulations accurately capture these results, where we classified behaviour as "cue" or "place" guided if the agent reached the platform as indicated by the landmark or previous location within a given number of time teps (60), and as "neither" otherwise.

**Deterministic two-step task.** In the experiment designed by Doll et al. (19), human participants where shown a pair of two pictures from one of two categories (faces or tools) and were asked to choose one. This was defined as the start state. The participants' initial choice determined which of two second-stage states they would transition to. These second stage states corresponded to a choice from a pair of pictures from one of two new categories (scenes or body parts; see Figure S3A). Each second-stage option (the 'outcome') was either rewarded with money or not rewarded. The reward probability for each outcome drifted slowly and randomly such that participants continuously learned by trial and error which second-stage choices were most likely to be rewarded. The total expected value of both scene and body part states was made equal to avoid inducing a bias. The first-stage choices deterministically led to different outcomes: selecting one of the tools or one of the faces always led to the scenes, while the other tool or face always led to the body parts.

This task structure dissociates behaviour consistent with MB and MF learning. A model-based learner represents transition probabilities, and uses this transition model to compute the best action. Thus, when a model-based learner encounters a reward, this should affect its behaviour in the next trial regardless of whether it starts in the same state as the previous trial (for example, faces followed by faces) or in a different one (for example, faces followed by tools). In contrast, a MF learner evaluates options in terms of the outcomes they have previously produced. Therefore, a model-free learner, upon receiving a reward, will only increase the probability of taking the same action in the next trial if that next trial starts in the same state as the previous one. Consistent with humans making use of both strategies, Doll and colleagues showed that human performance on this task lies somewhere in between these strategies (Figure S3B).

Our model recapitulates the main effects found by Doll and colleagues. The SR model mimics model-based behaviour by separating reward information from information about the transition structure. When the goal is reached, value is generalised to states that predict the goal states. Thus, following reward, the hippocampal model will learn to take actions to end up in the same second stage state in the next trial, regardless of whether it has the same or different starting state (Figure S3C). In contrast, the striatal learner learns separate action values for each state. Therefore, rewards obtained following one start state will not affect action values in the other start state (Figure S3C). Combining these two models gives a pattern of behaviour in between model-based and model-free, akin to human performance. However, in contrast to our model, human participants showed a higher stay probability for the "same starting state" condition than for the "different starting state" condition. This propensity to stay with the same action does not follow directly from a MF/MB trade-off.

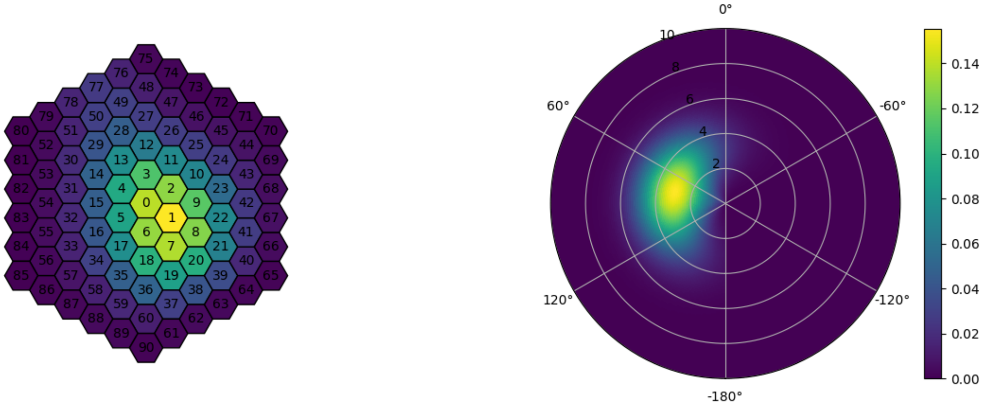


**Fig. S1.** Example receptive fields. Left panel: Example SR place cell map in a discretised maze. Right panel: Example landmark cell receptive field plotted in polar coordinates.

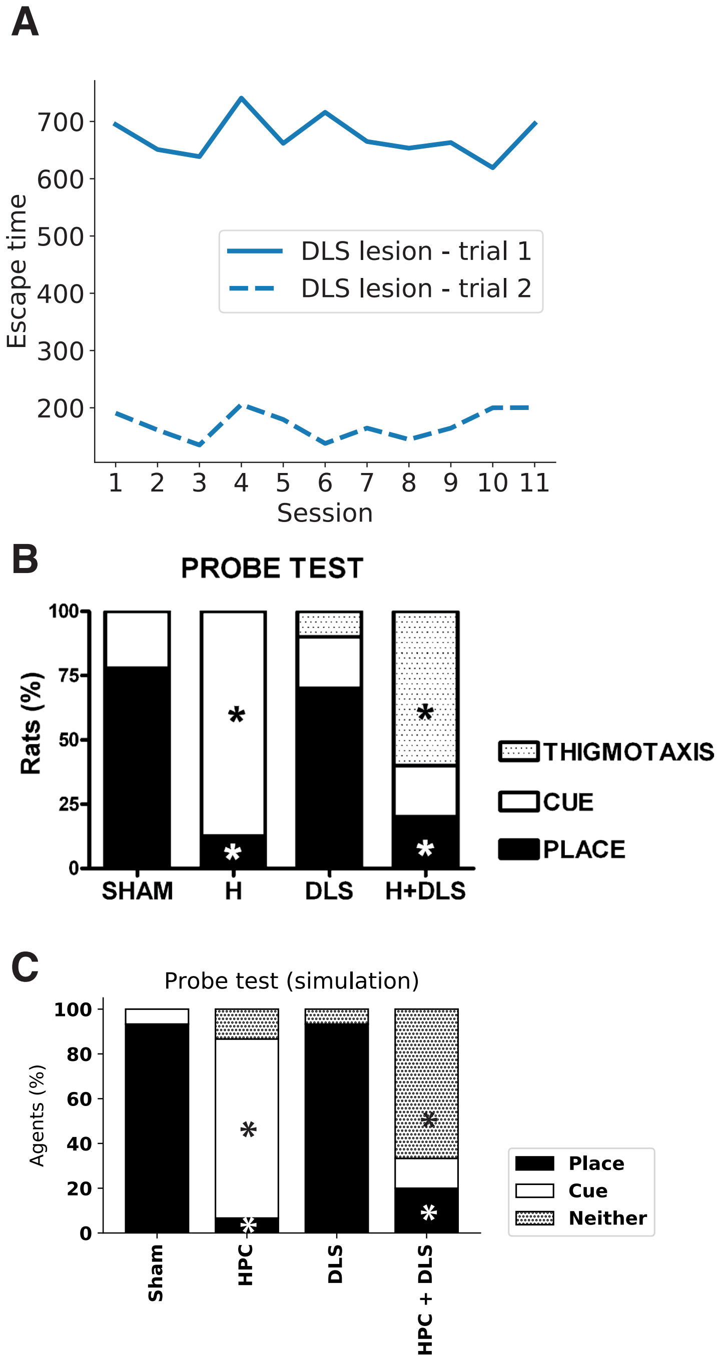


**Fig. S2.** (A) Simulation result of a DLS lesion in the Pearce et al. (16) study, showing escape time on the first and fourth trial of each session. Landmark and platform were moved together after every session. (B) Data from Myoshi et al. (18) showing the effects of bilateral lesions of the dorsal hippocampus (H) and/or the dorsolateral striatum (DLS) on a probe test carried out after 5 days of training on the Morris Water Maze. Data express the proportion of rats that (i) swam directly to the cued platform, (ii) to the place the hidden platform was the day before, or (iii) exhibited thigmotaxic swimming behaviour (swimming around the edges of the pool) in the first trial in the cued version. *P < 0.05 compared to SHAM animals; Fisher test. (C) Simulation results showing the effects of ablating the HPC and/or DLS model components on the task described in (B). * correspond to P < 0.05 in a Fisher test compared to SHAM animals/agents in both (B) and (C).

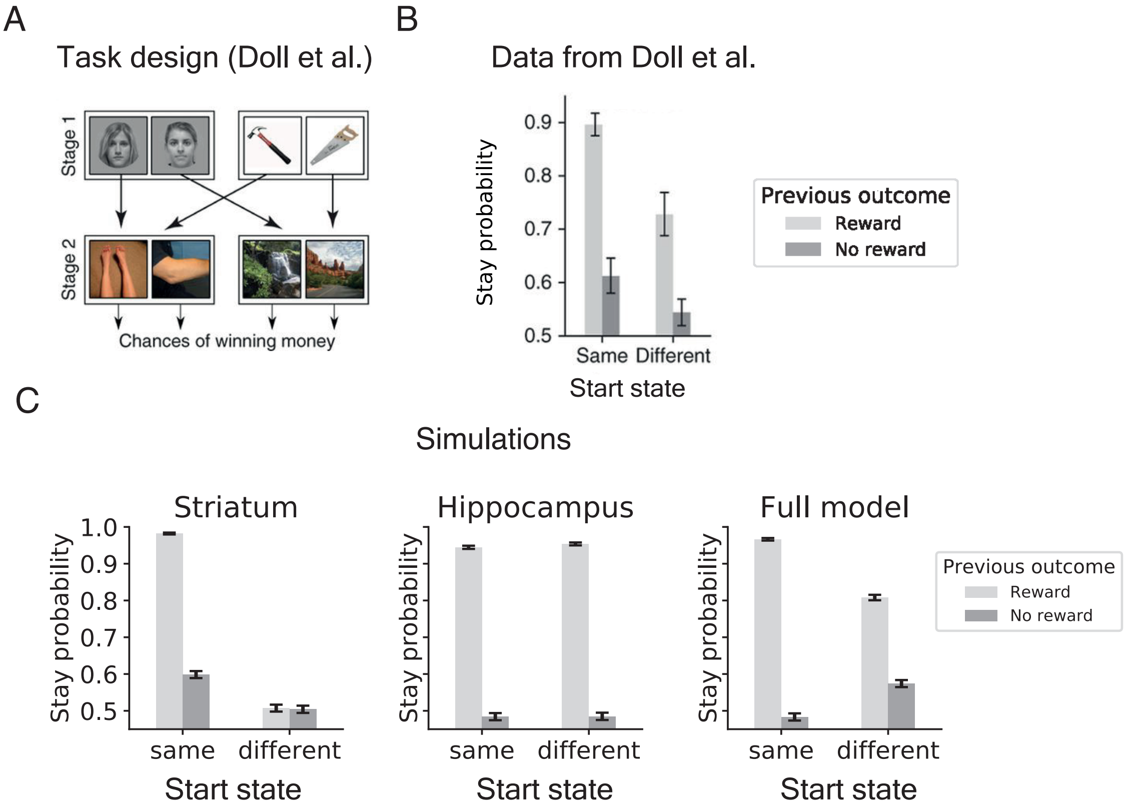


**Fig. S3.** (A) Task stucture employed by (19). (B) The probability that human participants in Doll et al. (2015) chose the same first-stage action as on the previous trial binned by whether the previous choice was rewarded, and whether they started in the same state. (C) Simulation results. The hippocampal model mimics the true MB agent presented in the original paper. The striatal model shows MF behaviour. Combining the two models results in a behavioural pattern that shows both effects. As in (19), MB behaviour was quantified as the main effect of previous reward on choice behaviour (estimate=.96, $Z = 4.3$, $P = 1.66 \times 10^{-5}$). This effect is greater when the current state is the same as the previous one (estimate=2.37, Z=6.64, $P = 3.18 \times 10^{-11}$), indicating the presence of MF behaviour.

| Name | Symbol | Value |
|---|---|---|
| SR learning rate | $\alpha_M$ | 0.07 |
| Q learning rate | $\alpha_Q$ | 0.07 |
| Softmax inverse temperature (exploration parameter) | $\tau^{-1}$ | 5 |
| Discount parameter | $\gamma$ | 0.95 |
| Reliability learning rate | $\eta$ | 0.03 |
| Maximum prediction error | $\delta_{MAX}$ | 1 |
| Steepness of transition curve MF to SR | $A_\alpha$ | 3.2 |
| Steepness of transition curve SR to MF | $A_\beta$ | 1.1 |

**Table S1. Parameters**

## References

1. S Wan Lee, S Shimojo, JP O'Doherty, Neural Computations Underlying Arbitration between Model-Based and Model-free Learning. *Neuron* **81**, 687–699 (2014).
2. A Barreto, R Munos, T Schaul, D Silver, Successor Features for Transfer in Reinforcement Learning. *arXiv*, 1–13 (2016).
3. N Burgess, A Jackson, T Hartley, J O'keefe, Predictions derived from modelling the hippocampal role in navigation. *Biol. cybernetics* **83**, 301–312 (2000).
4. T Hartley, N Burgess, C Lever, F Cacucci, J O'Keefe, Modeling place fields in terms of the cortical inputs to the hippocampus. *Hippocampus* **10**, 369–379 (2000).
5. C Barry, et al., The boundary vector cell model of place cell firing and spatial memory. *Rev. Neurosci.* **17**, 71–98 (2006).
6. RM Grieves, É Duvelle, PA Dudchenko, A boundary vector cell model of place field repetition. *Spatial Cogn. & Comput.* **18**, 217–256 (2018).
7. W de Cothi, C Barry, Neurobiological successor features for spatial navigation. *Hippocampus*, 1–9 (2020).
8. C Lever, S Burton, A Jeewajee, J O'Keefe, N Burgess, Boundary vector cells in the subiculum of the hippocampal formation. *J. Neurosci.* **29**, 9771–9777 (2009).
9. MAA van der Meer, A Johnson, NC Schmitzer-Torbert, AD Redish, Triple dissociation of information processing in dorsal striatum, ventral striatum, and hippocampus on a learned spatial decision task. *Neuron* **67**, 25–32 (2010).
10. OM Vikbladh, et al., Hippocampal Contributions to Model-Based Planning and Spatial Memory. *Neuron* **102**, 683–693.e4 (2019).
11. ND Daw, SJ Gershman, B Seymour, P Dayan, RJ Dolan, Model-based influences on humans' choices and striatal prediction errors. *Neuron* **69**, 1204–1215 (2011).
12. S Seabold, J Perktold, statsmodels: Econometric and statistical modeling with python in *9th Python in Science Conference*. (2010).
13. O Vikbladh, et al., Two Sides of the Same Coin: The Hippocampus as a Common Neural Substrate for Model-Based Planning and Spatial Memory. *bioRxiv* (2018).
14. RA Fisher, Frequency distribution of the values of the correlation coefficient in samples from an indefinitely large population. *Biometrika* **10**, 507–521 (1915).
15. R Core Team, *R: A Language and Environment for Statistical Computing* (R Foundation for Statistical Computing, Vienna, Austria), (2013) ISBN 3-900051-07-0.
16. JM Pearce, ADL Roberts, M Good, Hippocampal lesions disrupt navigation based on cognitive maps but not heading vectors. *Nature* **62**, 1997–1999 (1998).
17. AA Braun, et al., Dopamine depletion in either the dorsomedial or dorsolateral striatum impairs egocentric Cincinnati water maze performance while sparing allocentric Morris water maze learning. *Neurobiol. Learn. Mem.* **118**, 55–63 (2015).
18. E Miyoshi, et al., Both the dorsal hippocampus and the dorsolateral striatum are needed for rat navigation in the Morris water maze. *Behav. Brain Res.* **226**, 171–178 (2012).
19. BB Doll, KD Duncan, DA Simon, D Shohamy, ND Daw, Model-based choices involve prospective neural activity. *Nat. Neurosci.* **18**, 767–772 (2015).
